# Distinctive features of immunostaining and mutational load in primary pulmonary enteric adenocarcinoma: implications for differential diagnosis and immunotherapy

**DOI:** 10.1186/s12967-018-1449-z

**Published:** 2018-03-27

**Authors:** Ming Chen, Pu Liu, Feifei Yan, Suzhen Xu, Qi Jiang, Jingying Pan, Mengye He, Peng Shen

**Affiliations:** 1 Department of Medical Oncology, First Affiliated Hospital, Zhejiang University School of Medicine, 79 Qingchun Road, Hangzhou, 310003 People’s Republic of China; 2 Department of Pathology, First Affiliated Hospital, Zhejiang University School of Medicine, Hangzhou, 310003 China

**Keywords:** Primary pulmonary enteric adenocarcinoma, Immunohistochemistry, Tumor mutation burden, Targeted therapy, Immunotherapy

## Abstract

**Background:**

Primary pulmonary enteric adenocarcinoma (PEAC) is an extremely rare variant of invasive lung cancer. It is highly heterogeneous while shares some common morphologic and immunohistochemical features with usual pulmonary adenocarcinoma (PAC) and colorectal adenocarcinoma (CRAC), making the differential diagnosis difficult. At present there are only limited studies about distinctive features of primary PEAC and the results are often inconsistent.

**Methods:**

We retrospectively analyzed total 129 primary PEACs and 50 CRACs that were published since 1991 or diagnosed in our centre. Among them eight typical samples of primary PEACs and usual PACs were detected by targeted exome sequencing.

**Results:**

The combination of CK7^+^/CDX2^+^ acquires high sensitivity (71.3%) and specificity (82%) in differential diagnosis of PEACs from CRAC. The primary PEACs harbor a high incidence of *KRAS* mutation but almost absent of *EGFR* mutation. Moreover, compared with usual PACs, the primary PEACs have higher nonsynonymous tumor mutation burden and more frequent *MMR* mutation.

**Conclusions:**

The combination of CK7^+^/CDX2^+^ immunostaining and the distinctive genetic signatures, including low incidence of sensitivity genes mutations and high tumor mutation burden, is an important supplementary to the clinical differential diagnosis of primary PEACs. Our findings thus have significant implications for development of individualized treatment strategy in these patients.

## Background

Primary pulmonary enteric adenocarcinoma (PEAC) is an extremely rare variant of invasive lung adenocarcinoma. It was firstly described in 1991 by Tsao and Fraser [1], and categorized as an independent pathological subtypes in the International Multidisciplinary Classification of Lung Adenocarcinoma proposed by the International Association for the Study of Lung Cancer (IASLC)/American Thoracic Society (ATS)/European Respiratory Society (ERS) in 2011 [2]. But the relative diagnostic criteria were not determined until 2015 by World Health Organization (WHO) [3]. Based on these criteria, primary PEAC mainly (> 50%) composes of tall columnar cells with eosinophilic cytoplasm that arrange in irregular glandular cavities or cribriform pattern with central necrosis. For immunohistochemistry, primary PEAC expresses at least one of the enteric differentiation markers (CDX2, CK20, and MUC2). And in approximately half the cases, CK7 and TTF-1 are consistently positive.

Primary PEACs were highly heterogeneous and shared some morphologic and immunohistochemical appearances with pulmonary adenocarcinoma (PAC) and colorectal adenocarcinoma (CRAC). It could even present the typical patterns of colorectal cancer. The differential diagnosis between primary PEACs and metastatic CRAC was challenging but of important clinical implications, since it impacted on clinical stage, therapeutic strategy and prognosis seriously.

Of course, a circumspect analysis of clinical history, physical examinations (CT, FDG-PET or fiberoptic colonoscopy) and careful long-term follow-up to exclude the possibility of intestinal cancer metastasis is obviously necessary. Emerging studies analyzed the characteristics of immunohistochemistry and gene mutation profile in primary PEACs to assist the differential diagnosis and to explore new therapeutic targets [4, 5]. It was regrettable that all previous published studies were either single case report or of small series. Mainly due to its low morbidity, current studies about pathogenesis, clinical features and treatment strategy of primary PEAC are limited and the results are inconsistent. The distinctive immunohistochemical and genetic signature are still absent.

In our present study, we collected 18 samples of primary PEACs diagnosed in our centre and retrospectively reviewed 111 cases published since 1991, aimed at comprehensively analyzing the features of clinicopathology, immunohistochemistry and gene mutation profile of primary PEAC. Furthermore, compared with usual PACs, eight classic samples were chose to be analyzed for the genetic signature and tumor mutation burden (TMB) using targeted exome sequencing of 315 oncogenes and tumor suppressor genes.

## Methods

This work obtained each patients’ informed consent and was approved by the Research Ethic Committee in First Affiliated Hospital of Zhejiang University school of Medicine.

### Tumor selection

The cases of lung adenocarcinoma collected from 2008 to 2017 in the First Affiliated Hospital of Zhejiang University school of Medicine were screened according to the WHO 2015 criteria of primary PEAC. Two pathologists reviewed the specimens independently. After excluding possible colorectal cancer metastasis by carefully analyzing the clinical histories and imaging examinations, 18 samples of primary PEACs were enrolled in our study. Also, 50 samples of colorectal adenocarcinoma were collected randomly to analysis the immunohistochemical expression of CK7 and CDX2.

### Immunohistochemical analysis

The immunohistochemical analysis was conducted as previously described [6]. A panel of markers, including caudal type homeobox 2 (CDX2), cytokeratin 20 (CK20), cytokeratin 7 (CK7) and thyroid transcription factor-1 (TTF-1) were tested. The representative images were collected using Leica DM-2500 biological microscope, Germany.

### Gene mutation and tumor mutation burden (TMB) assessment

The gene mutation profiles in five classic samples of primary PEAC and three usual PAC samples that with no history of smoking were detected by targeted exome sequencing of 315 oncogenes and tumor suppressor genes using Illumina next seq 500 DNA sequencer, USA. The experimental procedure was performed as described previously [7]. Sequence data was processed using an analysis pipeline designed by YunYing Medical Technology Company to accurately detect multiple classes of genomic alterations: base substitutions, short insertions/deletions, copy-number alterations and selected gene fusions. Compare the sequence data between tumor tissue and the normal tissue, and then filter out background mutation in order to compute the TMB.

### Search strategy

A comprehensive search was performed through PubMed using the literature retrieval strategy “(pulmonary enteric adenocarcinoma [Title/Abstract]) OR Pulmonary adenocarcinoma with enteric differentiation [Title/Abstract]) OR Pulmonary intestinal-type adenocarcinoma [Title/Abstract]) OR lung enteric adenocarcinoma [Title/Abstract]) OR enteric-type adenocarcinoma of the lung [Title/Abstract]) OR Intestinal type of Lung Adenocarcinoma [Title/Abstract]) OR Pulmonary Adenocarcinoma With Intestinal Differentiation [Title/Abstract]” in January 2018 (no year limit and all languages included). Relevant articles were obtained, and references from each of these articles were further searched for relevant articles. A total of 43 articles were reviewed (of which 22 were case reports or series). There were a total of 129 reported cases from which data were collated and analyzed.

### Statistical analysis

Continuous variables were described with mean ± standard deviation (SD). Categorical data were described with percentage. For continuous variables, two independent samples t test was used to test the differences between the two groups. And for categorical data, Chi square test was used to test the differences between different groups. Sensitivity, specificity, 95% CI of them, and ROC analysis were performed to evaluate diagnostic value of CK7^+^/CDX2^+^ on primary PEACs. SPSS version 16.0 (SPSS Inc., Chicago, IL) software and GraphPad Prism 5.0 (GraphPad Software, San Diego, CA) was used for statistical analysis and graphics, respectively. The statistical significance level was 0.05.

## Results

### Clinicopathologic data

**Fig. 1:**
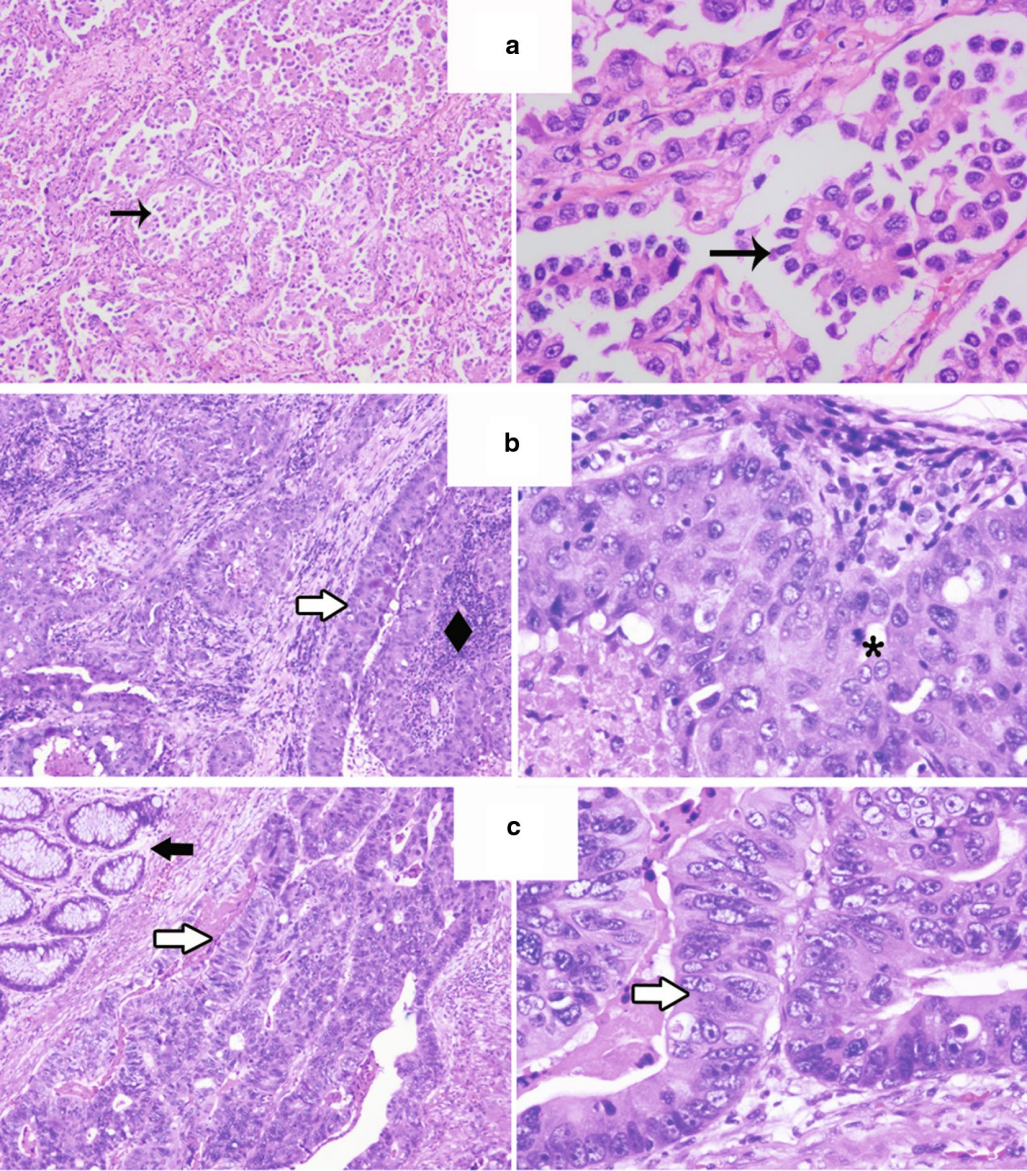
The representative images of the histopathology for usual PAC (**a**), primary PEAC (**b**) and CRAC (**c**) were shown. Primary PEACs consisted predominantly of enteric-type components that tall columnar cells arranged in irregular glandular cavities with central necrosis. Left, H&E, ×50; Right, H&E, ×200. *H&E* hematoxylin and eosin staining, *PAC* pulmonary adenocarcinoma, *PEAC* pulmonary enteric adenocarcinoma, *CRAC* colorectal adenocarcinoma. Rightwards arrow indicates the nipples of pulmonary adenocarcinoma; rightwards double arrow indicates the tumor cells were tall columnar cells with eosinophilic cytoplasm; black diamond suit indicates central necrosis; heavy concave pointed black rightwards arrow indicates the normal intestinal epithelium; asterisk indicates mitoses

Eighteen cases of primary PEAC diagnosed in our medical center from 2008 to 2017 were enrolled into this study and representative images of the histopathology for primary PEAC, usual PAC and CRAC is shown in Fig. 1. The most important clinical features of the cases were summarized in Table 1. Among them, 15/18 (83.3%) were represented by surgical specimens and 3/18 (16.7%) were biopsies. Among them, 6 patients were men, and 12 were women (the male/female ratio was 1:2). Patients were elderly with an average age of 63.2 years (range 55–76). 4/18 patients were cigarette smokers (22.2%). Three patients died within a follow-up period of 4–96 months. While for the tumor size and location, it seemed no specific.

**Table 1:** Clinicopathologic data

	No. (n/N) (%)
*Patients’ clinical characteristics (N = 18)*	
Age	
Median (range)	63.2 (55–76)
Age > 60 years	11/18 (61)
Sex. female	12/18 (67)
Smoking	4/18 (22)
Positive serum tumor markers	
CEA	6/18 (33)
CA199	9/18 (50)
CYFRA 21-1	2/18 (11)
NSE	0/18 (0)
Location	
RLL/RUL/RML	9/18 (50)
LLL/LUL	9/18 (50)
Size (cm)	
Median (range)	3.1 (1.1–6.6)
pTNM classification	
Stage I/II	12/18 (67)
Stage III/IV	6/18 (33)
Follow-up (M)	
Median (range)	31 (4–96)
Died	3/16 (19)
Alive	13/16 (81)
Not available	2/16 (12.5)

The clinical characteristics of 18 primary PEACs diagnosed in our medical center from 2008 to 2017 were summarized. The levels of these four tumor markers when patient first visit were compared with the upper limit of normal expression ranges, and the multiples were showed in table. The normal expression ranges of these four tumor markers: CEA, 0–5 μg/L; CA199, 0–37 U/mL; CYFRA21-1, 0–7 μg/L; NSE, 0–30 μg/L; All cases were staged according to the pathological tumor/node/metastasis (pTNM) classification (8th edition) of the IASLC

*M* male, *F* female, *CEA* carcinoembryonic antigen, *CA199* carbohydrate antigen, *CYFRA21-1* cytokeratin 19 fragment, *NSE* neuron-specific enolase, *RLL* right lower lobe, *RUL* right upper lobe, *LLL* left lower lobe, *LUL* left upper lobe, *RML* right middle lobe, *mo* month, *D* died, *A* alive, *NA* not available

We also analyzed the expression level of serum tumor markers, including CEA, CA199, NSE and CYFRA21-1, which were commonly used in clinic. Interestingly, our results showed that the expression of carcinoembryonic antigen (CEA) and carbohydrate antigen (CA199) was more remarkable than cytokeratin 19 fragment (CYFRA21-1) and neuron-specific enolase (NSE) in primary PEACs. CA199 was the mostly expressed marker while NSE expression was nearly absent.

### Immunohistochemical analysis

All primary PEACs studies that previously published concerning CDX2, CK20, CK7 and TTF-1 were retrospectively analyzed, and the immunohistochemical results were summarized in Table 2 [4, 5, 8–24]. As shown in the Table 2, for the intestinal markers, the positive percentage of CDX2 staining (79.1%) was much higher than that of CK20 (48.1%). And for pneumocytic markers, the positive CK7 staining (89.9%) was more remarkable than TTF-1 (40.3%). CK7 and CDX-2 were the immunohistochemical markers that mostly expressed in primary PEACs. The expression of CK7 and TTF-1 was positive consistently in approximately 36% of the PEAC cases.

**Table 2:** Review all the studies concerning IHC markers of primary PEACs

Study	No. of cases	Immunohistochemical results			
		CK-7	TTF-1	CK-20	CDX2
Yousem [8]	6	6+	6+	6−	6−
Inamura et al. [9]	7	7+	3+/4−	3+/4−	5+/2−
Li et al. [10]	1	1−	1−	1+	1+
Hatanaka et al. [11]	1	1−	1−	1+	1+
Maeda et al. [12]	1	1+	1+	1−	NA
Lin et al. [13]	1	1+	1−	1+	1−
Qureshi et al. [14]	1	1+	1−	1+	1+
Wang et al. [15]	9	9+	4+/5−	2+/7−	6+/3−
Stojsic et al. [16]	2	2−	2−	2+	2+
Laszlo et al. [17]	1	1−	1−	1+	1+
Garajová et al. [18]	2	1+/1−	2−	1+/1−	2+
Metro et al. [19]	1	1+	1−	1−	1+
Handa et al. [20]	1	1+	1+	1−	1−
Nottegar et al. [4]	46	46+	21+/25−	15+/31−	46+
Lin et al. [21]	1	1−	1−	1+	1+
Matsushima et al. [22]	8	7+/1−	1+/7−	7+/1−	5+/3−
Nottegar et al. [5]	8	8+	1+/7−	1+/7−	8+
Sun et al. [23]	1	1+	1−	1−	1+
Bian et al. [24]	13	10+/3−	7+/6−	8+/5−	8+/5−
The present study	18	16+/2−	7+/11−	17+/1−	13+/5−
Total	129	116+/13−	52+/77−	62+/67−	102+/26−
Positive expression (%)		89.9	40.3	48.1	79.1

The studies concerning immunohistochemical analysis of primary PEAC that published since 1991 were retrospectively reviewed. The information of CDX2, CK20, CK7 and TTF-1 expression in these studies was collected and their rates of positive expression were computed

*IHC* immunohistochemical, *TTF-1* thyroid transcription factor 1, *CK* cytokeratin, *CDX2* caudal type homeobox 2, *NA* not available

**Fig. 2:**
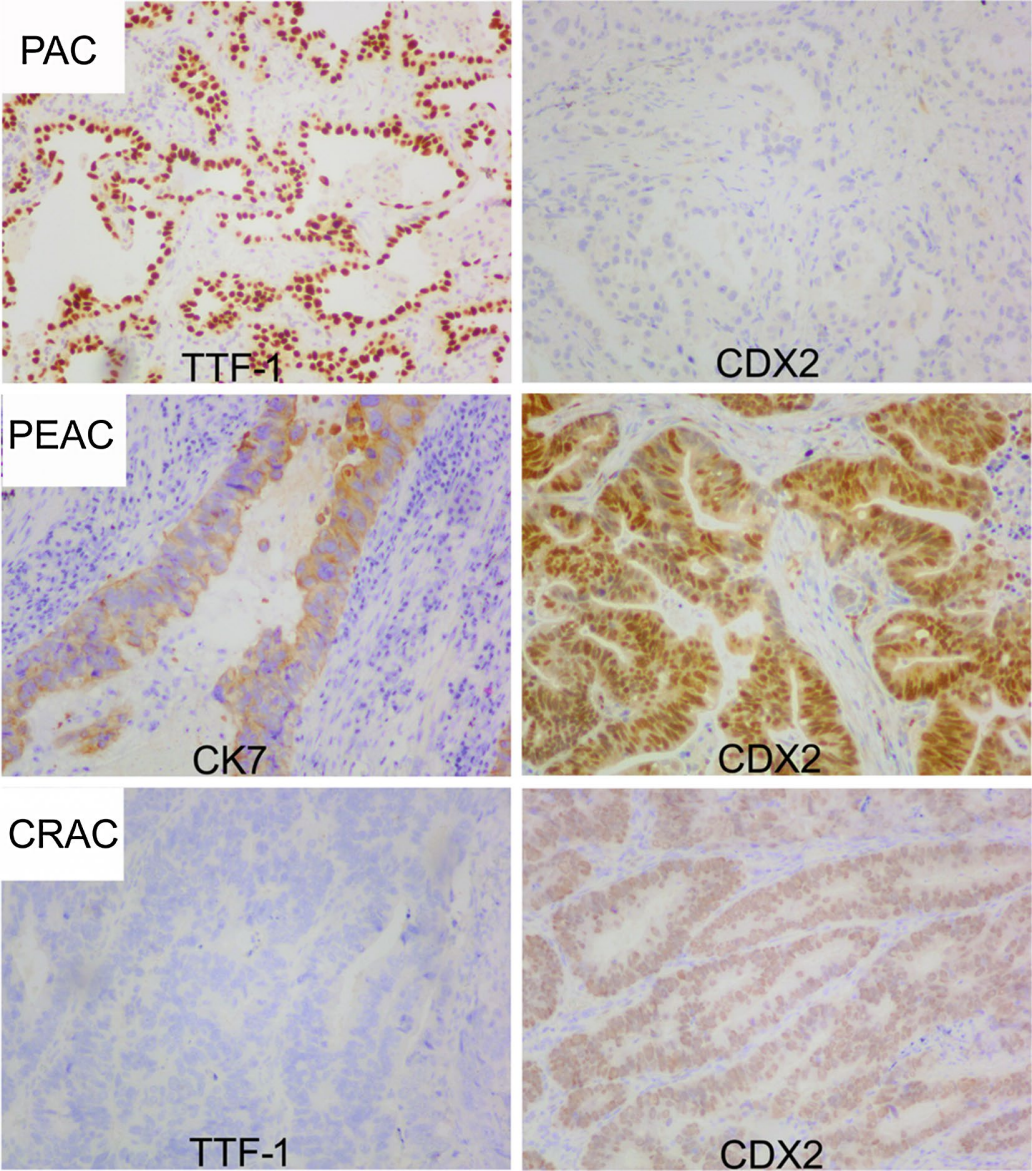
The representative images of the IHC markers immunostaining for usual PAC, primary PEAC and CRAC were shown. H&E, ×100. *H&E* hematoxylin and eosin staining, *PAC* pulmonary adenocarcinoma, *PEAC* pulmonary enteric adenocarcinoma, *CRAC* colorectal adenocarcinoma, *CK* cytokeratin, *TTF‑1* thyroid transcription factor 1, *CDX2* caudal type homeobox 2

Moreover, we also analyzed the expression of CK7 and CDX2 in 50 samples of CRAC that collected randomly in our centre. Our results suggested that the combination of CK7^+^/CDX2^+^ acquired high sensitivity (71.3, 95% CI 63.5–79.1%) and specificity (82%, 95% CI 71.4–92.6%) in the differential diagnosis of primary PEACs from CRACs, as shown in Table 3. Additionally, ROC analysis also suggested well diagnostic value of CK7^+^/CDX2^+^ on Primary PEACs (area, 0.767, 95% CI 0.689–0.844, P < 0.01). The representative images of the immunostaining for usual PAC, primary PEAC and CRAC were shown in Fig. 2 (usual PAC: TTF-1^+^, CDX2^−^; primary PEAC: CK7^+^, CDX2^+^; CRAC: TTF-1^−^, CDX2^+^; TTF-1 was chosen because of its better specificity than CK7 in the diagnosis for lung adenocarcinoma).

**Table 3:** The immunostaining of CK7^+^/CDX2^+^ in primary PEACs and CRACs

	No. of cases	CK7^+^/CDX2^+^	Non-(CK7^+^/CDX2^+^)
Primary PEACs	129	92 (71.3%)	37 (29%)
CRACs	50	9 (18%)	41 (82%)

The expression of CK7 and CDX2 was analyzed in 129 cases of primary PEAC and 50 cases of colorectal carcinoma. The consistently positive expression of CK7 and CDX2 acquired high sensitivity (71.3, 95% CI 63.5–79.1%) and specificity (82, 95% CI 71.4–92.6%) in the differential diagnosis of primary PEAC from CRACs. ROC analysis also suggested well diagnostic value of CK7^+^/CDX2^+^ on primary PEACs (area, 0.767, 95% CI 0.689–0.844, P < 0.01)

### Gene mutation and TMB analysis

All studies concerning gene abnormalities in primary PEACs were analyzed [4, 5, 15–22]. As shown in Table 4, almost half of the cases (47.6%) harbored *KARS* gene mutation in exon 2, 3 and 4. The incidence of *EGFR* gene, *NRAS* gene and *EML4*-*ALK* fusion mutations was extremely low (3.7, 7.7 and 9.9% respectively), and the *BRAF* gene was wild type in all cases.

**Table 4:** Review all the studies concerning mutational analysis of primary PEACs

Study	No. of cases	Gene mutation						
		*KARS* exon2-4	*EGFR* exon 18-21	*EML4*-*ALK*	*BRAF* exon15	*NRAS* exon2-4	*ERBB2 (HER2)* amplification/mutation	*MMR*
Stojsic et al. [16]	2	1+/1−	2−	NA	NA	NA	NA	NA
Lászlo et al. [17]	1	1+	1−	NA	NA	NA	NA	NA
Garajová et al. [18]	2	2+	2−	2−	NA	NA	NA	NA
Metro et al. [19]	1	1+	NA	NA	NA	NA	NA	NA
Lin et al. [21]	1	1−	1−	1−	1−	NA	NA	NA
Wang et al. [15]	9	9−	9−	9−	NA	NA	NA	NA
Handa et al. [20]	1	NA	1+	NA	NA	NA	NA	NA
Nottegar et al. [4]	46	28+/18−	1+/45−	6+/40-	46−	NA	NA	NA
Matsushima et al. [22]	7	1+/6−	7−	NA	7−	NA	NA	NA
Nottegar et al. [5]	8	4+/4−	8−	1+/7−	8−	8−	NA	NA
The present study	5	1+/4−	1+/4−	5−	5−	1+/4−	2+/3−	4+/1−
Total		39+/43−	3+/79−	7+/64−	67−	1+/12−	2+/3−	4+/1−
Incidence of mutation (%)		47.6	3.7	9.9	0	7.7	40	80

The studies concerning sensitivity genes mutation in primary PEAC that published since 1991 were reviewed. Our results from targeted exome sequencing of 315 CGPs in five classic samples of primary PEAC were also showed

**Fig. 3:**
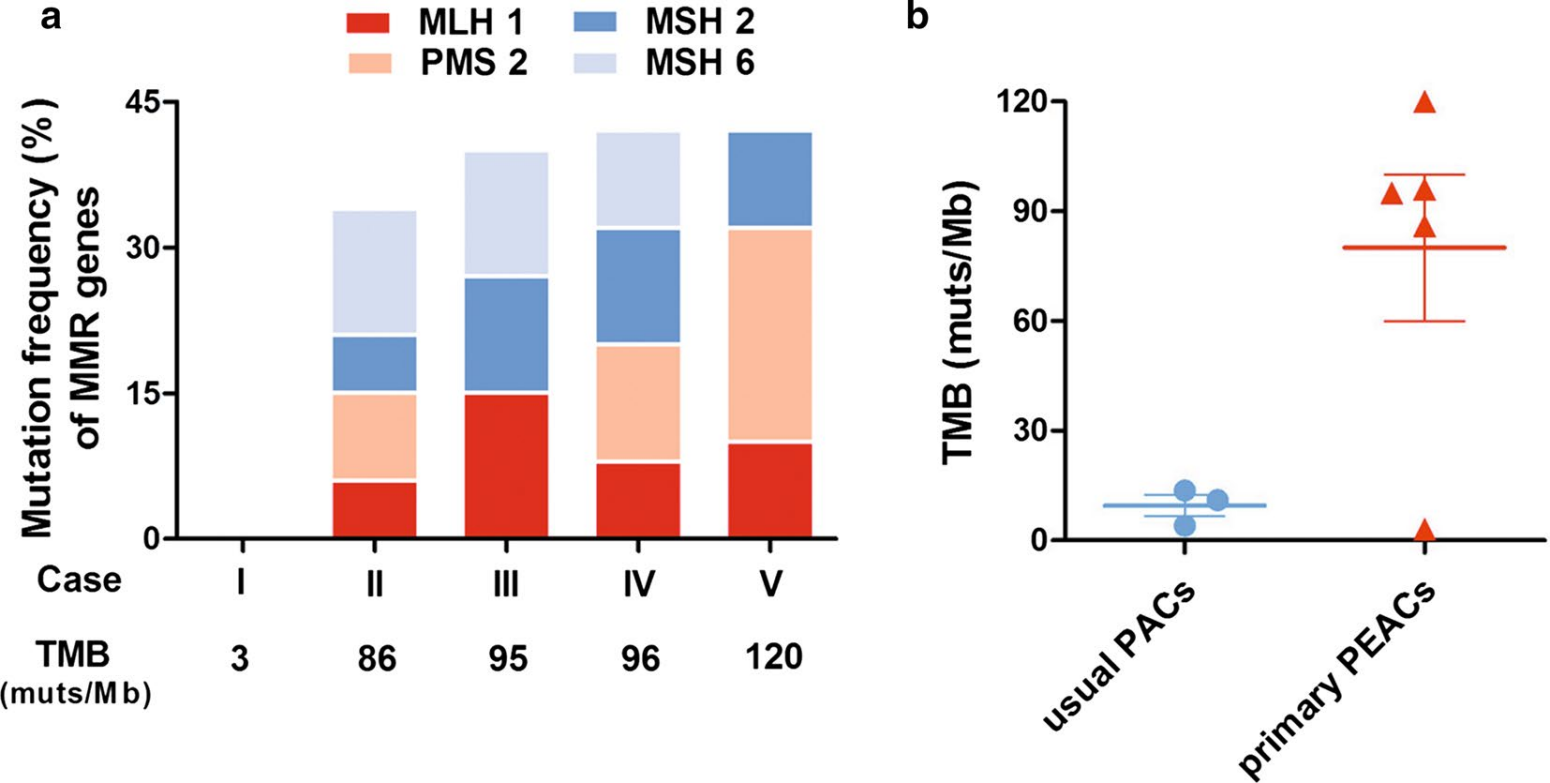
Primary PEACs harbored a high incidence of *MMR* genes mutation and higher TMB compared with usual PACs. **a** 4/5 primary PEAC patients harbored *MMR* genes mutation and the mutation frequency of core *MMR* genes (*MLH1, PMS2, MSH2 and MSH6*) and the TMB (muts/Mb) of each patient was showed. **b** The TMB of primary PEACs (mean: 80.0 ± 20.1 muts/Mb, N = 5) was significantly higher than that of usual PACs (mean: 9.5 ± 2.9 muts/Mb, N = 3) (t = − 2.627, P (2-tailed) = 0.039, P < 0.05)

Five classic cases of primary PEAC with no smoking history were chose to be further analyzed for gene mutation by targeted exome sequencing of 315 oncogenes and tumor suppressor genes. Interestingly, we found the abnormalities of *ERBB2 (HER2)* and *MMR* genes. 2/5 (40%) harbored *ERBB2 (HER2)* amplification or mutation, while *MMR* genes showed mutation in 4/5 cases (80%) (Table 4). The mutation frequency of the core *MMR* genes (*MLH1, PMS2, MSH2 and MSH6*) and the TMB (muts/Mb) of each case was described in Fig. 3a. More importantly, as shown in Fig. 3b, the TMB (muts/Mb) in primary PEACs was significantly higher than that in usual PACs (mean: 80.0 ± 20.1 VS 9.5 ± 2.9, t = − 2.627, P (2-tailed) = 0.039, P < 0.05).

## Discussion

Primary PEAC is a special and rare type of lung adenocarcinoma. To make a definite diagnosis, the distinctive features of immunohistochemistry and gene mutation profile have been attracting more and more attention. By analyzing the cases diagnosed in our medical center, and retrospectively reviewing all published cases, a deeper understanding of primary PEAC is widely expected.

As for the clinical features of primary PEAC, we find that primary PEACs are more occurred in elderly women rather than younger patients. Moreover, it is interesting to find that the levels of serum tumor markers CEA and CA199 expression are increased more remarkably than that of CYFRA21-1 and NSE. Of these markers, CA199 is the mostly expressed while NSE expression is nearly absent. Furthermore, the consistent overexpression of CEA and CA199 seems to be closely-related with advanced pathologic stage in primary PEACs. As we known, CEA is nonspecific to diagnose a variety of cancers, such as colon cancer, lung cancer, breast cancer and so on. The high serum expression of CA199 is more predictive for the digestive tract tumors, like colorectal cancer and pancreatic cancer. But CYFRA21-1 and NSE are more specific and sensitive to the diagnosis of lung cancer (non-small cell lung cancer, small cell lung cancer respectively). Our results reveal that different from usual lung cancer, the serum expression levels of tumor marker CEA and CA199 should be given more attention in the diagnosis, therapeutic monitoring and relapse prediction of primary PEAC, although more studies are needed to validate it.

Immunohistochemical marker is significant to the pathological diagnosis, especially in primary PEAC, since it is not enough based solely on its morphologic features. Thus many studies had focused on the immunohistochemical signatures of primary PEACs. The study of Yousem et al. [8] indicated that immunohistochemical markers of primary PEACs were associated with respiratory tract rather than enteric canal. Inamura et al. [9] proposed that CK7 and CK20 could be used as markers for distinction of PEACs from metastatic colorectal carcinomas. Nottegar et al. [4] analyzed the immunohistochemical results of 46 PEACs and considered that CDX-2 and CK7 positivity was very robust to support the diagnosis of PEAC. Two studies also explored the usefulness of SATB2, β-catenin or CDH17 immunostaining in the differentiation diagnosis between PEACs and metastatic colorectal carcinoma [22, 24]. In general, the results were inconsistent, and the value of the studies was limited by the relatively small series of cases. Although it was mentioned in the 2015 WHO criteria that primary PEAC expressed at least one of the enteric differentiation markers (CDX2, CK20 and MUC2), a distinctive immunohistochemical signature of primary PEAC with much clinical applicability to differentiate from colorectal cancer was still lack.

As the most typical and clinical routinely detected immunohistochemical markers of pneumocytic and intestinal, CK7, TTF-1, CK20 and CDX2 were chose to be analyzed in the present study. In total 129 cases of primary PEAC, CK7 and CDX-2 were the markers that mostly expressed. It was consistent with the previous study by Nottegar et al. [4]. Moreover, our results showed that the expression of CK7 and TTF-1 was consistently positive in approximately 36% of the PEAC cases which was less than the previous report [3]. Since it exhibited immunoreactivity for both pulmonary- and intestinal-type markers, the diagnosis of primary PEAC could not relied on a certain immunohistochemical marker. Our study suggested that the combination of CK7^+^/CDX2^+^ acquired high sensitivity and specificity in the differential diagnosis from colorectal carcinoma, which showed great application value in clinical practice. Considering the high heterogeneity of histomorphology, the combination of CK7^+^/CDX2^+^ exhibited much more importance in diagnosis of primary PEAC.

To validate the molecular profiles, almost all studies concerning mutational information of primary PEAC were retrospectively analyzed. Consistent with the results of the study by Nottegar et al. [4], we also found that the mutations of exon2, 3 and 4 in *KRAS* gene were much more prevalent than *EGFR*, *NRAS* genes and *EML4*-*ALK* fusion gene in primary PEACs, while the mutation of *BRAF* gene was total absent. Our results above suggested that using EGFR tyrosine kinase inhibitors in primary PEAC patients might be unreasonable and inefficient, which was different from that in usual lung adenocarcinoma.

Immunotherapy is considered as a major breakthrough as cancer treatment in recent years, and its efficacy had been confirmed in a variety of tumor types, like advanced NSCLC, melanoma, kidney cancer and so on. In advanced NSCLC, Reck et al. [25] demonstrated that treatment with pembrolizumab was associated with significantly longer PFS, OS and fewer adverse events than platinum-based chemotherapy in patients with PD-L1 expression on at least 50% of tumor cells. Although immunotherapy could be highly effective, only a minority of patients acquired good response to it. So identifying patients who were most likely to benefit from these therapies was very important. Of course, measurement of PD-1/PD-L1 expression was technically feasible, but the immunostaining of PD-L1 was limited by the lack of unified standard and was difficult to interpret [26]. TMB, defined as the number of nonsynonymous mutations in the tumor, such as nonsynonymous base substitutions, short insertions/deletions, copy-number alterations and selected gene fusions, was emerging to act as a predictive marker associated with response to checkpoint blockade immunotherapy [27–29]. The study of Rizvi et al. [28] revealed a significant association between TMB and the sensitivity to PD-1 blockade in NSCLC patients. Their results suggested that higher nonsynonymous TMB was associated with the improved objective response, durable clinical benefit, and PFS of pembrolizumab.

For the first time, using targeted exome sequencing of 315 oncogenes and tumor suppressor genes, we detected the TMB of primary PEACs and compared it with usual pulmonary adenocarcinomas. To our surprise, the nonsynonymous TMB of primary PEACs was significantly higher than that of usual pulmonary adenocarcinomas, which TMB was low in all three cases we detected. Thus we conjectured that checkpoint blockade immunotherapy might be a new light in primary PEAC patients. In view of the possible role absence of tyrosine kinase inhibitors in primary PEACs, the news that these patients might have greater possibility to benefit from checkpoint blockade immunotherapy was exciting, especially for those advanced patients, although further studies were needed to ascertain this. The complications associated with checkpoint blockade in primary PEAC patients, including immune-related adverse events (irAEs), were also worthy of our attention.

Interestingly, we found that *ERBB2 (HER2)* amplification/mutation and *MMR* genes mutation could also be occurred in primary PEACs. 2/5 primary PEACs harboring the amplification/mutation of *ERBB2 (HER2)* gene reminded that certain patients with this rare variation of pulmonary adenocarcinoma might be responsive to the therapy targeted at *HER2* gene product, like herceptin and pertuzumab, or the inhibitors of HER2 tyrosine kinase, like afatinib. The *MMR* genes mutation might impair the base mismatch repair system and resulted in the genome instability. Many studies had validated the correlation between MMR deficiency and the response to checkpoint blockade immunotherapy [27, 28, 30]. In the present study, 4/5 patients of primary PEAC exhibited *MMR* genes mutation. Furthermore, the mutational status of the core *MMR* genes, *MLH1*, *PMS2*, *MSH2 and MSH6*, was consistent with the level of TMB in each sample we tested. Similar to the findings in other studies, the loss of DNA MMR enzymes was associated with a high TMB [27, 31]. But it should be noted that the efficacy of immunotherapy in our study was not validated because of the absent survival data. The association of high TMB and *MMR* genes mutation with immunotherapy in primary PEACs still needs to be checked.

## Conclusions

The combination of CK7^+^/CDX2^+^ immunostaining and the distinctive genetic signatures, including low incidence of sensitivity genes mutations and high tumor mutation burden, provides important supplementary information to clinical differential diagnosis of primary PEACs and has significant implications in individualized treatment strategy in these patients. Our study suggest that, different from it in usual lung adenocarcinoma, EGFR tyrosine kinase inhibitor used in primary PEACs might be unreasonable and inefficient, but these patients might have greater possibility to benefit from checkpoint blockade immunotherapy.

## Abbreviations

PEAC: pulmonary enteric adenocarcinoma
PAC: pulmonary adenocarcinoma
CRAC: colorectal adenocarcinoma
IASLC: International Association for the Study of Lung Cancer
ATS: American Thoracic Society
ERS: European Respiratory Society
WHO: World Health Organization
TMB: tumor mutation burden
CGPs: cancer gene panels
CDX2: caudal type homeobox 2
CK20: cytokeratin 20
CK7: cytokeratin 7
TTF-1: thyroid transcription factor-1
CEA: carcinoembryonic antigen
CA199: carbohydrate antigen
CYFRA21-1: cytokeratin 19 fragment
NSE: neuron-specific enolase

## References

[1] Tsao MS, Fraser RS (1991). Primary pulmonary adenocarcinoma with enteric differentiation. Cancer.

[2] Travis WD, Brambilla E, Noguchi M, Nicholson AG, Geisinger K, Yatabe Y, Powell CA, Beer D, Riely G, Garg K (2011). International Association for the Study of Lung Cancer/American Thoracic Society/European Respiratory Society: international multidisciplinary classification of lung adenocarcinoma: executive summary. Proc Am Thorac Soc.

[3] Travis WD, Brambilla E, Burke AP, Marx A, Nicholson AG (2015). Introduction to the 2015 World Health Organization classification of tumors of the lung, pleura, thymus, and heart. J Thorac Oncol.

[4] Nottegar A, Tabbo F, Luchini C, Brunelli M, Bria E, Veronese N, Santo A, Cingarlini S, Gilioli E, Ogliosi C (2016). Pulmonary adenocarcinoma with enteric differentiation: immunohistochemistry and molecular morphology. Appl Immunohistochem Mol Morphol.

[5] Nottegar A, Tabbo F, Luchini C, Guerrera F, Gaudiano M, Bria E, Brunelli M, Chilosi M, Inghirami G (2017). Pulmonary adenocarcinoma with enteric differentiation: dissecting oncogenic genes alterations with DNA sequencing and FISH analysis. Exp Mol Pathol.

[6] Luchini C, Capelli P, Fassan M, Simbolo M, Mafficini A, Pedica F, Ruzzenente A, Guglielmi A, Corbo V, Scarpa A (2014). Next-generation histopathologic diagnosis: a lesson from a hepatic carcinosarcoma. J Clin Oncol.

[7] Frampton GM, Fichtenholtz A, Otto GA, Wang K, Downing SR, He J, Schnall-Levin M, White J, Sanford EM, An P (2013). Development and validation of a clinical cancer genomic profiling test based on massively parallel DNA sequencing. Nat Biotechnol.

[8] Yousem SA (2005). Pulmonary intestinal-type adenocarcinoma does not show enteric differentiation by immunohistochemical study. Mod Pathol.

[9] Inamura K, Satoh Y, Okumura S, Nakagawa K, Tsuchiya E, Fukayama M, Ishikawa Y (2005). Pulmonary adenocarcinomas with enteric differentiation: histologic and immunohistochemical characteristics compared with metastatic colorectal cancers and usual pulmonary adenocarcinomas. Am J Surg Pathol.

[10] Li HC, Schmidt L, Greenson JK, Chang AC, Myers JL (2009). Primary pulmonary adenocarcinoma with intestinal differentiation mimicking metastatic colorectal carcinoma: case report and review of literature. Am J Clin Pathol.

[11] Hatanaka K, Tsuta K, Watanabe K, Sugino K, Uekusa T (2011). Primary pulmonary adenocarcinoma with enteric differentiation resembling metastatic colorectal carcinoma: a report of the second case negative for cytokeratin 7. Pathol Res Pract.

[12] Maeda R, Isowa N, Onuma H, Miura H (2008). Pulmonary intestinal-type adenocarcinoma. Interact Cardiovasc Thorac Surg.

[13] Lin D, Zhao Y, Li H, Xing X (2013). Pulmonary enteric adenocarcinoma with villin brush border immunoreactivity: a case report and literature review. J Thorac Dis.

[14] Qureshi A, Furrukh M (2013). Enteric adenocarcinoma lung: a rare presentation in an Omani woman. BMJ Case Rep.

[15] Wang CX, Liu B, Wang YF, Zhang RS, Yu B, Lu ZF, Shi QL, Zhou XJ (2014). Pulmonary enteric adenocarcinoma: a study of the clinicopathologic and molecular status of nine cases. Int J Clin Exp Pathol.

[16] Stojsic J, Kontic M, Subotic D, Popovic M, Tomasevic D, Lukic J (2014). Intestinal type of lung adenocarcinoma in younger adults. Case Rep Pulmonol.

[17] Laszlo T, Lacza A, Toth D, Molnar TF, Kalman E (2014). Pulmonary enteric adenocarcinoma indistinguishable morphologically and immunohistologically from metastatic colorectal carcinoma. Histopathology.

[18] Garajova I, Funel N, Fiorentino M, Agostini V, Ferracin M, Negrini M, Frassineti GL, Gavelli G, Frampton AE, Biasco G, Giovannetti E (2015). MicroRNA profiling of primary pulmonary enteric adenocarcinoma in members from the same family reveals some similarities to pancreatic adenocarcinoma-a step towards personalized therapy. Clin Epigenetics.

[19] Metro G, Valtorta E, Siggillino A, Lauricella C, Cenci M, Ludovini V, Minenza E, Prosperi E, Ricciuti B, Rebonato A (2015). Enteric-type adenocarcinoma of the lung harbouring a novel KRAS Q22K mutation with concomitant KRAS polysomy: a case report. Ecancermedicalscience.

[20] Handa Y, Kai Y, Ikeda T, Mukaida H, Egawa H, Kaneko M (2016). Pulmonary enteric adenocarcinoma. Gen Thorac Cardiovasc Surg.

[21] Lin LI, Xu CW, Zhang BO, Liu RR, Ge FJ, Zhao CH, Jia RU, Qin QH, Stojsic J, Wang Y, Xu JM (2016). Clinicopathological observation of primary lung enteric adenocarcinoma and its response to chemotherapy: a case report and review of the literature. Exp Ther Med.

[22] Matsushima J, Yazawa T, Suzuki M, Takahashi Y, Ota S, Nakajima T, Yoshino I, Yokose T, Inoue T, Kawahara K, Nakatani Y (2017). Clinicopathological, immunohistochemical, and mutational analyses of pulmonary enteric adenocarcinoma: usefulness of SATB2 and beta-catenin immunostaining for differentiation from metastatic colorectal carcinoma. Hum Pathol.

[23] Sun WW, Xu ZH, Wang CF, Wu F, Cao JM, Cui PJ, Huang W, Jin XL, Li B, Chen KM (2017). Pulmonary enteric adenocarcinoma with pancreatic metastasis: a case report. Oncol Lett.

[24] Bian TT, Zhao JL, Feng J, Zhang Q, Qian L, Liu J, Jiang DS (2017). Combination of cadherin-17 and SATB homeobox 2 serves as potential optimal makers for the differential diagnosis of pulmonary enteric adenocarcinoma and metastatic colorectal adenocarcinoma. Oncotarget.

[25] Reck M, Rodriguez-Abreu D, Robinson AG, Hui R, Csoszi T, Fulop A, Gottfried M, Peled N, Tafreshi A, Cuffe S (2016). Pembrolizumab versus chemotherapy for PD-L1-positive non-small-cell lung cancer. N Engl J Med.

[26] Topalian SL, Taube JM, Anders RA, Pardoll DM (2016). Mechanism-driven biomarkers to guide immune checkpoint blockade in cancer therapy. Nat Rev Cancer.

[27] Le DT, Uram JN, Wang H, Bartlett BR, Kemberling H, Eyring AD, Skora AD, Luber BS, Azad NS, Laheru D (2015). PD-1 blockade in tumors with mismatch-repair deficiency. N Engl J Med.

[28] Rizvi NA, Hellmann MD, Snyder A, Kvistborg P, Makarov V, Havel JJ, Lee W, Yuan J, Wong P, Ho TS (2015). Cancer immunology. Mutational landscape determines sensitivity to PD-1 blockade in non-small cell lung cancer. Science.

[29] Snyder A, Makarov V, Merghoub T, Yuan J, Zaretsky JM, Desrichard A, Walsh LA, Postow MA, Wong P, Ho TS (2014). Genetic basis for clinical response to CTLA-4 blockade in melanoma. N Engl J Med.

[30] Le DT, Durham JN, Smith KN, Wang H, Bartlett BR, Aulakh LK, Lu S, Kemberling H, Wilt C, Luber BS (2017). Mismatch repair deficiency predicts response of solid tumors to PD-1 blockade. Science.

[31] Stadler ZK, Battaglin F, Middha S, Hechtman JF, Tran C, Cercek A, Yaeger R, Segal NH, Varghese AM, Reidy-Lagunes DL (2016). Reliable detection of mismatch repair deficiency in colorectal cancers using mutational load in next-generation sequencing panels. J Clin Oncol.

